# Methodological rigour and reporting quality of the literature on wildlife rescue, rehabilitation, and release: a global systematic review

**DOI:** 10.1080/01652176.2025.2478138

**Published:** 2025-04-07

**Authors:** Gloeta N. Massie, Louis J. Backstrom, Daniel P. Holland, Mandy B.A. Paterson, Richard A. Fuller

**Affiliations:** a School of the Environment, The University of Queensland, St Lucia, QLD, Australia; b School of Mathematics and Statistics, The University of St Andrews, St Andrews, United Kingdom; c Independent researcher, Redlands, CA, USA; d School of Veterinary Science, The University of Queensland, Gatton, QLD, Australia; e RSPCA QLD, Wacol, QLD, Australia

**Keywords:** Wildlife rescue, wildlife rehabilitation, wildlife release, methodological rigour, reporting quality, systematic review, ARRIVE guidelines, animal welfare

## Abstract

Wildlife rescue, rehabilitation, and release is a global practice with a broad body of scientific literature; nonetheless, no studies have assessed and quantified the methodological rigour and reporting quality of this literature. In this PRISMA systematic review, we assessed and quantified the reporting of controls, randomisation, blinding, experimental animal data, and housing and husbandry data in 152 primary studies on wildlife rescue, rehabilitation, and release published between 1980 and 2021. We then tested for associations between reporting and study characteristics. Of the 152 reviewed studies, one study reported a control, randomisation, and blinding; 17 studies reported species, age, sex, weight, and body condition; and 14 studies reported housing size, housing location, type of food, provision of water, and provision of enrichment. No study reported all 13 of these elements. Studies published in veterinary-focused journals reported lower methodological rigour and had lower reporting quality than studies published in other types of journals. Studies on mammals had higher reporting quality than studies on birds and on reptiles, and studies that included the word “welfare” had higher reporting quality than studies that did not. The overall low methodological rigour and reporting quality of the literature limits study replicability and applicability and impedes meta-analyses.

## 1. Introduction

Wildlife rescue, rehabilitation, and release (hereafter wildlife rescue) is a global practice that impacts hundreds of thousands (Pyke and Szabo 2018) to potentially millions of wild animals annually (Cope et al. 2022), with implications for both conservation (Pyke and Szabo 2018; Simeone et al. 2024) and animal welfare (Mullineaux 2014; Willette et al. 2023). While both the number of animals rescued and the volume of scientific literature on wildlife rescue are increasing each year, previous studies have noted a lack of methodological rigour and reporting quality in the literature (Mullineaux 2014; Pyke and Szabo 2018; Cope et al. 2022; Simeone et al. 2024). However, no studies have systematically quantified these shortcomings. This is of concern because, as highlighted in numerous reviews on research involving animals more broadly (Carbone and Austin 2016; Percie Du Sert et al. 2020), weak methodological rigour and low reporting quality can result in bias (Altman and Bland 1999), reduced replicability (Kafkafi et al. 2018), and overstated outcomes and conclusions (Bebarta et al. 2003; Kang et al. 2008). Any of these issues could lead to increased animal resource costs (e.g. lack of statistical power due to insufficient sample sizes requiring repeating the experiment using additional animals with the attendant procurement and maintenance costs) (Percie Du Sert et al. 2020) and decreased animal welfare, including causing unnecessary pain, injury, or even death (Carbone and Austin 2016).

One method of assessing and quantifying the methodological rigour and reporting quality of the scientific literature is through the application of metrics such as the ARRIVE (Animals in Research: Reporting *In Vivo* Experiments) guidelines (hereafter Guidelines) (Kilkenny et al. 2010; Leung et al. 2018; Mbuagbaw et al. 2020; Percie Du Sert et al. 2020). Using the Guidelines, one can capture a snapshot of the state of the literature and explore changes in methodological rigour and reporting quality across factors such as time, journal of publication, study location, and study species (Leung et al. 2018; Calderón-Amor et al. 2023). In addition to those factors, in this review we also analysed whether the simple inclusion of the word ‘welfare’ in a study was associated with differences in methodological rigour and reporting quality. While evaluating animal welfare may not be a prime objective of a study, the welfare of the study animals during a study can directly impact a study’s outcomes (Percie Du Sert et al. 2020). Therefore, reporting on the factors that could have impacted animal welfare, regardless of a study’s aims, is critical to producing robust, replicable science (Percie Du Sert et al. 2020).

The aims of this systematic review were to (i) quantify the methodological rigour of the scientific literature on wildlife rescue, (ii) quantify the reporting quality of experimental data in the scientific literature on wildlife rescue, and (iii) test for associations between aims (i) and (ii) and study characteristics. To our knowledge, this is the first application of the Guidelines to the literature on wildlife rescue and the first analysis of whether the inclusion of the word ‘welfare’ in a study is associated with differences in methodological rigour and reporting quality across any animal study.

## 2. Methods

We followed the PRISMA (Preferred Reporting Items for Systematic reviews and Meta-Analyses) 2020 guidelines (Page et al. 2021) with modifications to account for our focus on methodological rigour and reporting quality (Murad and Wang 2017). We did not register a protocol review. A completed PRISMA 2020 checklist is available in Online Appendix A.

### 2.1. Searches

#### 2.1.1. Preliminary searches

We generated a list of preliminary welfare-focused search terms based on Duke University’s sample keywords for searches on animal welfare (Duke University, n.d.). To avoid species-biasing the results, we chose to use broad animal categories (e.g. we searched for ‘bird’ and not ‘*Buteo buteo*’ or ‘common buzzard’). We iteratively conducted preliminary searches between March 2020 and April 2021 in SCOPUS, Web of Science, Google Scholar, and PubMed to refine the search term list. Due to the lack of ‘near’ search syntax capabilities within the PubMed and Google Scholar syntaxes, however, we decided to narrow our searches solely to SCOPUS and Web of Science.

#### 2.1.2. Final search

GM conducted the final search in SCOPUS and Web of Science on 8 April 2021. The search syntaxes for the database queries and the number of results returned for each search are in Online Appendix B. Search phrasings included combinations of the words: wildlife, mammal, bird, amphibian, reptile, centre, shelter, clinic, hospital, medical, carer, oiled, rescu*, rehab*, post-releas*, orphan*, analgesi*, anesthes*, euthanas*, improv*, handl*, hous*, cag*, non-invasive, monitor*, behav*, ‘positive reinforcement’, welfare, adverse, pain, suffer, stress, distress, harm, mortality, outcome, and was limited to articles and reviews. Both SCOPUS and Web of Science automatically searched for lemmatisations and English-variants.

#### 2.1.3. Deduplication of results

GM deduplicated the search results using SR-Accelerator (Clark et al. 2020), Covidence (Covidence systematic review software 2021), and Zotero (version 5.0.96), since no single software captured all duplicates.

### 2.2. Screening

Two blinded reviewers (GM and MP) completed the title and abstract screening and the full text screening using Covidence, set to require double-blinding and review of conflicts. Initial ten percent random literature screens were used to validate and revise the protocols as necessary. All screening conflicts were discussed and resolved and both reviewers agreed on the final screening results.

#### 2.2.1. Exclusion criteria

We excluded any study that: was not a primary study; was not in English; did not include at least one wild-born animal rescued from the wild due to injury, illness, or being orphaned; did not describe an explicit intervention and impact or post-release monitoring; or did not intend to return the animal(s) to the wild. We also excluded any study where the full text was not retrievable through the University of Queensland library.

#### 2.2.2. Inclusion criteria

We included any type of study (e.g. case study, case control study, cohort study, randomised controlled trial, etc.) that was a primary study in English including at least one wild-born animal removed from the wild due to injury, illness, or being orphaned, with the intent to return the animal to the wild, and that described either a specific intervention and impact or post-release monitoring.

### 2.3. Data extraction

Three blinded extractors (GM, LB, DH) completed the data extraction in Covidence using a custom-template modified from Leung et al. (2018). As of the 2020 update, the Guidelines (Percie Du Sert et al. 2020) contains 21 items; for our study we specifically focused on five of the Guidelines items. To determine the extent to which evidence for the practice of wildlife rescue was based on randomised, controlled, blinded experiments, we have analysed data from items 1 (study design, specifically the use of a control), 4 (randomisation), and 5 (blinding). To analyse the reporting quality of the types of universal data (i.e. not species-specific) necessary to analyse animal welfare, we assessed and analysed items 8 (experimental animals) and 15 (housing and husbandry). Table 1 shows the selected Guidelines items and subitems, the extraction questions to extract specific elements of those items and subitems, and the possible extractor responses.

**Table 1.** Data extraction table showing the ARRIVE guidelines 2.0 items and subitems used for data extraction, the extraction questions, and the possible extractor responses (Leung et al. 2018; Percie Du Sert et al. 2020).

ARRIVE guideline 2.0 item and, if relevant, subitem(s)	Extraction question	Possible extractor responses
*Item 1: study design* Subitem 1a: use of a control	Did the study report a control group?	Not reported; reported
*Item 2: sample size* Subitem 2a: total sample size	Did the study report a total sample size? If reported, what were the total number of experimental animals used in the study?	Not reported; reported, total number of experimental animals reported
*Item 4: randomisation* Subitem 4a: use of randomisation	Did the study report any usage of randomisation?	Not reported; reported
*Item 5: blinding*	Did the study report any usage of study blinding?	Not reported; reported
*Item 8: experimental animals* Sub-items 8a and 8b: provision of specific details about the study animals	Did the study report the species of any experimental animals? If yes, what types of species were reported?	Not reported; reported, type(s) of species reported
	Did the study report the sex of any experimental animals?	Not reported; reported
	Did the study report the age of any experimental animals?	Not reported; reported
	Did the study report the weight of any experimental animals?	Not reported; reported
	Did the study report the body condition of any experimental animals?	Not reported; reported
*Item 15: housing and husbandry*	Did the study report the size of the housing used to house any experimental animals?	Not reported; reported
	Did the study report whether any experimental animals were housed indoors or outdoors?	Not reported; reported
	Did the study report the type of food provided to any experimental animals?	Not reported; reported
	Did the study report the provision of water to any experimental animals?	Not reported; reported
	Did the study report the provision of enrichment to any experimental animals?	Not reported; reported

The extractors would select ‘reported’ if an element was reported for even a single animal anywhere in the study, i.e. extractors did not limit their data extraction to a study’s methods section. For example, an extractor would mark the weight element as reported if the study reported the weight of one animal in its discussion section, even if there were other animals in the study with no reported weights.

As with the screenings, Covidence was set to require double-blinding and review of conflicts. Ten percent random literature data extractions were used to validate and revise the extraction protocol as necessary. All extraction conflicts were discussed and resolved, and all extractors agreed on the final extraction results.

To build comparative groups, data were extracted on year of publication, journal of publication, study site location, species of experimental animals, sample size (subsequently excluded from analyses), and inclusion of the word ‘welfare’ in the main body of the study. These data were extracted using textual extraction, e.g. ‘2020; Journal of Wildlife; Gatton, Queensland, Australia; *Threskiornis molucca;* 7; included’. If the extractors were unable to locate any textual data or the textual data were unclear, the extractors would note this as ‘not reported or unclear’.

Results of the data extraction process are provided in Online Appendix C.

### 2.4. Statistical analyses

We grouped data for statistical analyses following Leung et al. (2018) and based on authors’ consensus. Groupings are shown in Table 2.

**Table 2.** Description of groupings for statistical analyses.

Variable	Method of grouping	Group names shown in analyses
Year of publication	As the original ARRIVE Guidelines were published in 2010 (Kilkenny et al. 2010), we divided the studies into two groups based on year of publication: 1980–2010 and 2011–2021	1980–2010; 2011–2021
Journal of publication	Studies were divided into two groups based on the name of the journal of publication. If the name of the journal included either the word veterinary (or a derivation thereof) or medicine (or a derivation thereof), it was grouped into ‘veterinary’; all other journals were grouped into ‘other’	Veterinary; Other
Study site location	After calculating the frequency of study sites per country, we divided the studies into three analytical groupings based on the total number of study sites in each location: USA (*n* = 60); Australia, South Africa, United Kingdom (*n* = 46); and all other countries (*n* = 46)	USA; Australia, South Africa, United Kingdom; All other countries
Class of study species	Studies were divided into three groups based on the taxonomic class of the study species	Aves (birds), Mammalia (mammals), and Reptilia (reptiles)
Inclusion of the word ‘welfare’	Studies were divided into two groups based on whether the study included the word ‘welfare’ anywhere in the main body of the study	Included; Not included

We used Microsoft Excel (version 2307) and SPSS (version 29.0.0.0 (241)) to organise and analyse data. We used R studio (version 4.2.1), the R package ‘ComplexUpset’ (version 1.3.3) (Lex et al. 2014; Krassowski 2020), and coding assistance from OpenAI (2024) to generate the UpSet plots. We used Adobe Illustrator (version 29.0 (64-bit) to modify the plots. We used the R package ‘taxize’ (version 0.1.100) with function ‘tax_name’ and databases ‘itis’ and ‘ncbi’ to confirm species’ classes, e.g.: ‘tax_name(sci = “Pelecanus occidentalis californicus”, get = “class”, db = “itis”)’ (Chamberlain and Szöcs 2013). All data necessary to replicate our statistical analyses are available in Online Appendix C.

Categorical data were analysed in SPSS using double-sided Fisher’s Exact Tests with the time limit per test set at five minutes. Our independent variables were: year of publication, journal of publication, study site location, class, and inclusion of the word ‘welfare’. Our dependent variables were: control, randomisation, blinding, class, sex, age, weight, body condition, housing size, housing location, type of food, provision of water, and provision of enrichment. We reported all results with *p* < .05 as significant and *p* ≥ .05 as non-significant.

## 3. Results

### 3.1. Search and screening results

**Figure 1.**
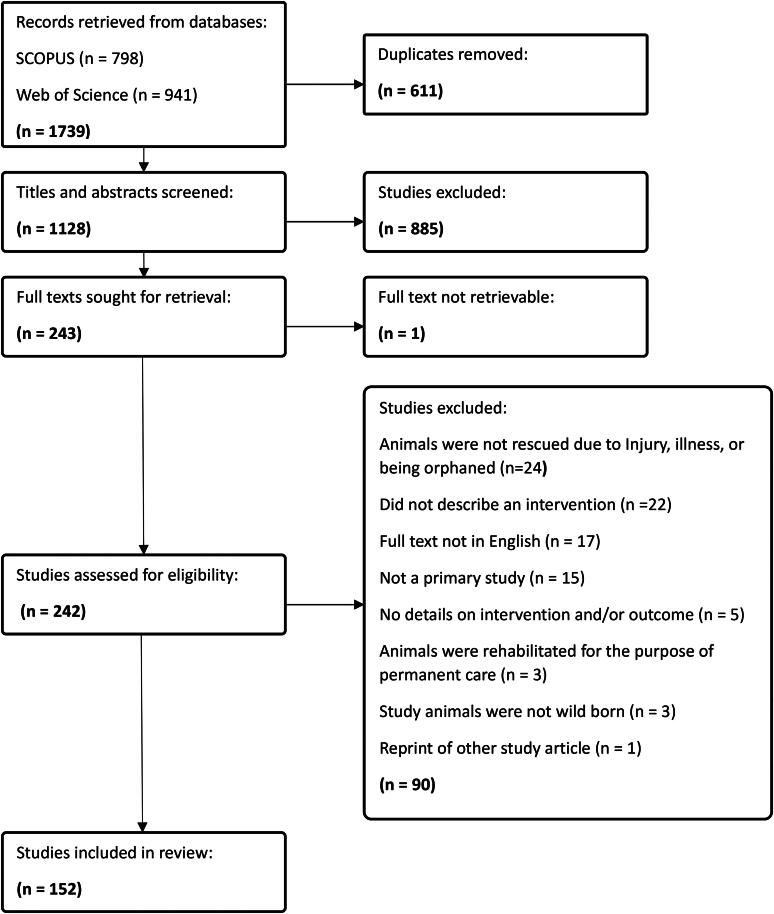
Diagram of search and screening processes and results.

The PRISMA flowchart is shown in Figure 1. The initial database search returned 1739 results. After de-duplication, title and abstract screening, and full text screening, 152 studies were included for data extraction.

#### 3.1.1. Title and abstract screening

Covidence calculated the proportionate agreement between GM and MP for the title and abstract screening as 0.90 and Cohen’s Kappa as 0.78.

#### 3.1.2. Full text screening

Covidence calculated the proportionate agreement between GM and MP for the full text screening as 0.89 and Cohen’s Kappa as 0.64.

### 3.2. Analytical results

#### 3.2.1. Aim (i) quantify the methodological rigour of the scientific literature on wildlife rescue

**Figure 2.**
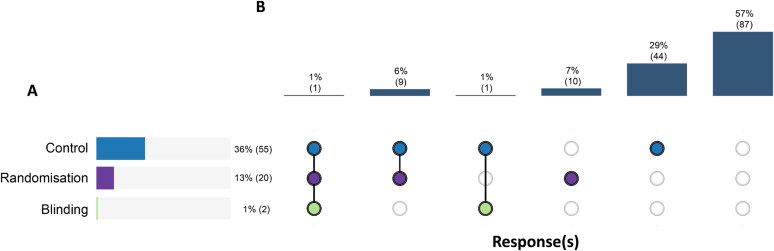
Number of studies reporting the use of a control, randomisation and blinding. (A) The horizontal bars show the percentage (frequency) of 152 studies that reported use of a control, randomisation, or blinding, with each element assessed independently. (B) The vertical bars show the percentage (frequency) of studies that reported specific combinations, represented by the columns of circles. A filled circle represents the reporting of the particular element and a hollow circle represents the absence of reporting of a particular element. From left to right, the combinations are ordered by the number of elements reported (three to zero) and by the element order in (a).

Reporting rates for the three methodological rigour elements (control, randomisation, and blinding) ranged from 1% (2/152) to 36% (55/152) of studies (Figure 2(A)). Only one study (1/152, 1%) reported use of a control, randomisation, and blinding, whereas 87 studies (87/152, 57%) reported none of these elements (Figure 2(B)). The mean and median number of methodological elements reported were 0.51 and 0, respectively.

#### 3.2.2. Aim (ii) quantify the reporting quality of experimental data in the scientific literature on wildlife rescue

**Figure 3.**
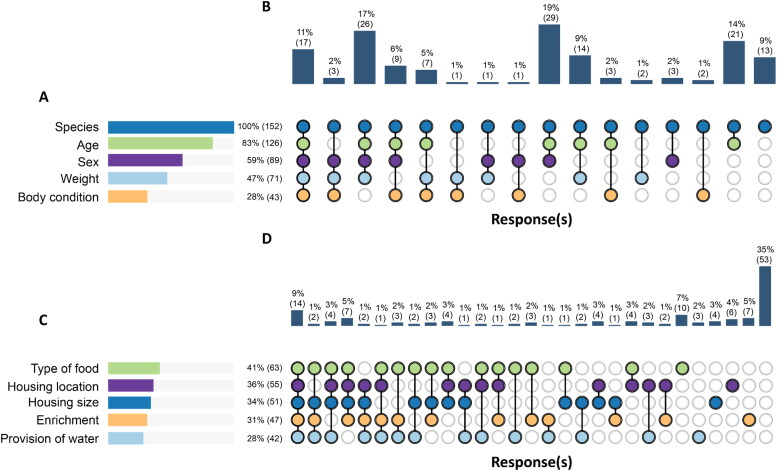
Number of studies reporting ARRIVE 2.0 elements related to experimental animals and housing and husbandry. The horizontal bars show the percentage (frequency) of 152 studies that reported a specific element related to either experimental animals (A) or housing and husbandry (C), with each element assessed independently. The vertical bars show the percentage (frequency) of studies that reported specific combinations of elements, represented by the columns of circles. A filled circle represents the reporting of the particular element and a hollow circle represents the absence of reporting of a particular element. From left to right, the combinations are ordered by the number of elements reported (five to zero) and by the element order in (a) or (C).

Reporting rates for the five individual experimental animal elements (species, sex, age, weight, and body condition) ranged from 28% (43/152) to 100% (152/152) of studies (Figure 3(A)). While 11% (17/152) of studies reported all five elements for experimental animals, 27% (41/152) of studies reported two or fewer elements (Figure 3(B)). The mean and median number of experimental animal elements reported were 3.16 and 3, respectively.

Reporting rates for the five individual housing and husbandry elements (housing size, housing location, type of food, provision of water, and enrichment) ranged from 28% (42/152) to 41% (63/152) of studies (Figure 3(C)). While 9% (14/152) of studies reported all five housing and husbandry elements, 70% (106/152) of studies reported two or fewer housing and husbandry elements (Figure 3(D)). Of note, 35% (53/152) of studies reported none of the five housing and husbandry elements. The mean and median number of housing and husbandry elements reported were 1.70 and 1, respectively.

Across the ten combined reporting elements of experimental animals and housing and husbandry, the mean and median number of elements reported were 4.86 and 5, respectively. Only one study (1/152, 1%) reported data on all ten experimental elements; conversely, six studies (6/152, 4%) did not report data on any experimental element other than species. Sixty-five percent (99/152) of studies reported five or fewer experimental elements.

#### 3.2.3. Aim (iii) test for associations between aims (i) and (ii) and study characteristics

A summary of study characteristics is provided in Table 3. Data are provided in Online Appendix C.

**Table 3.** Summary characteristics of the reviewed studies (*n* = 152).

Category	Summary
Year of publication	
Range of years of publication	1980–2021
Number of studies published per year: mean, median, and minimum-maximum	4, 2, 0–15
Year with greatest number of publications	2020, (15/152, 10%)
Years with zero publications	1981–1990; 1992, 1997–1998, 2009
Journal of publication	
Number of journals with reviewed studies	66 journals
Number of studies published per journal: mean, median, and minimum-maximum	2.30, 1, 1–20
Journal with the greatest number of reviewed studies	*Journal of Wildlife Rehabilitation*, (20/152, 13%)
Number of journals publishing only one study	40 journals
Study site location	
Number of study sites and countries	149 study sites in 27 countries
Number of study sites per country: mean, median, and minimum-maximum	6, 1, 1–58
Country with the greatest number of study sites	USA, (58/149, 39%)
Number of countries with only one study site	15 countries
Study species	
Total number of species reported in reviewed studies	342 species
Number of species per study: mean, median, and minimum-maximum	2.25, 1, 1–33
Number of unique species reported in reviewed studies	184 species
Taxonomic breakdown of species	3 classes
	34 orders
	61 families
	126 genera
Species included in the greatest number of studies	Harbor seal, *Phoca vitulina*, (8/152, 5%)
Number of species included in only one study	108 species

The frequency and percentage of reporting by groupings and the *p*-values from the double-sided Fisher Exact tests are shown in Table 4.

**Table 4.** Results of descriptive and inferential statistical analyses by subgroups. Data are presented as the number of studies reporting/the total number of studies in the subgroup (percentage of studies reporting by subgroup). A double-sided Fisher’s Exact test was conducted for each ARRIVE 2.0 subitem element and analytical grouping except species. Statistically significant *p*-values (*p* < .05) are in bold.

ARRIVE 2.0 subitem	Study characteristics										Class of study species				Inclusion of the word ‘welfare’		
	Year of publication			Journal of publication			Study site location										
	1980–2010	2011–2021	*p*-value	Veterinary	Other	*p*-value	USA	Australia, South Africa, United Kingdom	All other countries	*p*-value	Aves (birds)	Mammalia (mammals)	Reptilia (reptiles)	*p*-value	Included	Not included	*p*-value
Methodological rigour																	
Control	19/55 (35%)	36/97 (37%)	.86	7/40 (18%)	48/112 (43%)	**.004**	23/60 (38%)	17/46 (37%)	15/46 (33%)	.87	33/80 (41%)	20/65 (31%)	2/7 (29%)	.41	16/46 (35%)	39/106 (37%)	.86
Randomisation	9/55 (16%)	11/97 (11%)	.46	4/40 (10%)	16/112 (14%)	.59	10/60 (17%)	5/46 (11%)	5/46 (11%)	.69	9/80 (11%)	11/65 (17%)	0/7 (0%)	.49	7/46 (15%)	13/106 (12%)	.61
Blinding	0/55 (0%)	2/97 (2%)	.54	0/40 (0%)	2/112 (2%)	1	1/60 (2%)	0/46 (0%)	1/46 (2%)	1.00	2/80 (3%)	0/65 (0%)	0/7 (0%)	.55	2/46 (4%)	0/106 (0%)	.09
Experimental animals																	
Species	All subgroups had 100% reporting																
Sex	34/55 (62%)	55/97 (57%)	.61	23/40 (58%)	66/112 (59%)	1	31/60 (52%)	31/46 (67%)	27/46 (59%)	.28	34/80 (43%)	52/65 (80%)	3/7 (43%)	**< .001**	32/46 (70%)	57/106 (54%)	.08
Age	47/55 (85%)	79/97 (81%)	.66	29/40 (73%)	97/112 (87%)	.05	51/60 (85%)	36/46 (78%)	39/46 (85%)	.64	61/80 (76%)	60/65 (92%)	5/7 (71%)	**.01**	44/46 (96%)	82/106 (77%)	**.005**
Weight	29/55 (53%)	42/97 (43%)	.31	19/40 (48%)	52/112 (46%)	1	26/60 (43%)	21/46 (46%)	24/46 (52%)	.65	33/80 (41%)	35/65 (54%)	3/7 (43%)	.31	22/46 (48%)	49/106 (46%)	.86
Body condition	16/55 (29%)	27/97 (28%)	1.00	14/40 (35%)	29/112 (26%)	.31	16/60 (27%)	19/46 (41%)	8/46 (17%)	**.04**	26/80 (33%)	16/65 (25%)	1/7 (14%)	.45	16/46 (35%)	27/106 (25%)	.25
Housing and husbandry																	
Housing size	24/55 (44%)	27/97 (28%)	.05	7/40 (18%)	44/112 (39%)	**.01**	19/60 (32%)	17/46 (37%)	15/46 (33%)	.86	19/80 (24%)	30/65 (46%)	2/7 (29%)	**.02**	22/46 (48%)	29/106 (27%)	**.02**
Housing location	18/55 (33%)	37/97 (38%)	.60	14/40 (35%)	41/112 (37%)	1	25/60 (42%)	16/46 (35%)	14/46 (30%)	.52	26/80 (33%)	29/65 (45%)	0/7 (0%)	**.03**	23/46 (50%)	32/106 (30%)	**.03**
Type of food	26/55 (47%)	37/97 (38%)	.31	11/40 (28%)	52/112 (46%)	**.04**	26/60 (43%)	16/46 (35%)	21/46 (46%)	.52	23/80 (29%)	38/65 (58%)	2/7 (29%)	**< .001**	24/46 (52%)	39/106 (37%)	.11
Provision of water	17/55 (31%)	25/97 (26%)	.57	6/40 (15%)	36/112 (32%)	**.04**	23/60 (38%)	6/46 (13%)	13/46 (28%)	**.01**	17/80 (21%)	22/65 (34%)	3/7 (43%)	.12	12/46 (26%)	30/106 (28%)	.85
Enrichment	27/55 (49%)	20/97 (21%)	**< .001**	9/40 (23%)	38/112 (34%)	.23	20/60 (33%)	14/46 (30%)	13/46 (28%)	.88	22/80 (28%)	24/65 (37%)	1/7 (14%)	.37	16/46 (35%)	31/106 (29%)	.57

There were no significant associations between the reporting of randomisation or blinding and any analytical grouping. By contrast, there was a significant association between the reporting of a control and the journal of publication (p = .004). Only 18% of studies (7/40) published in veterinary-focused journals reported controls compared to 43% of studies (48/112) published in other types of journals.

There were no significant associations between the reporting of weight and any analytical grouping. By contrast, there were significant associations between: sex by class of study species (*p* = < .001), age by class of study species (*p* = .01), age by inclusion of the word ‘welfare’ (*p* = .005), and body condition by study site location (*p* = .04). Studies on mammals had higher reporting levels for sex and age than studies on birds and on reptiles, studies that included the word ‘welfare’ had higher reporting levels for age than studies that did not include the word ‘welfare’, and studies based in Australia, the United Kingdom, and South Africa had higher reporting levels for body condition than studies based in the USA and in all other countries.

There were significant associations between every reported element of housing and husbandry and analytical groupings: housing size by journal of publication (*p* = .01), housing size by class of study species (*p* = .02), housing size by inclusion of the word ‘welfare’ (*p* = .02), housing location by class of study species (*p* = .03), housing location by inclusion of the word ‘welfare’ (*p* = .03), type of food by journal of publication (*p* = .04), type of food by class of study species (*p* ≤ .001), provision of water by journal of publication (*p* = .04), provision of water by study site location (*p* = .01), and provision of enrichment by year block (*p* ≤.001). Similar to the reporting on experimental animal elements, studies focused on mammals had higher reporting levels for housing size and type of food than studies on birds and on reptiles, studies published in veterinary-focused journals had lower reporting levels on housing size, type of food, and the provision of water compared to studies published in other types of journals, and studies published between 2011–2021 had lower reporting levels on the provision of enrichment than studies published between 1980–2010.

## 4. Discussion

In this systematic review, we analysed the methodological rigour and reporting quality of the scientific literature on wildlife rescue and we tested for associations between methodological rigour, reporting quality, and study characteristics. Our analyses revealed limited methodological rigour, low reporting quality, geographic and taxonomic biases, and multiple barriers to improvement.

### 4.1. Methodological rigour

Although blinded, randomised controlled trials are considered the gold-standard of interventional research, only one study included in this review reported blinding, randomisation, and a control. While we recognise that large-scale, blinded, randomised controlled trials are outside of the capacity of many interventional settings, the use of methodologically rigorous designs should be the rule, not the exception. Even single-animal studies, for example, can be blinded, randomised, and controlled through the use of methods such as personnel blinding, randomisation generators, and washout periods (Percie Du Sert et al. 2020). We strongly urge researchers to pursue the most methodologically rigorous studies possible, within practical and ethical boundaries, as weak methodologies generate weak evidence, and weak evidence limits the improvement of wildlife rescue.

### 4.2. Reporting quality

Wildlife rescue is theoretically predicated on improving the welfare of the rescued wildlife (Mullineaux 2014; Englefield et al. 2019; Willette et al. 2023). Therefore, we expected studies on rescued wildlife to report the types of data previously shown to influence animal welfare (Percie Du Sert et al. 2020), regardless of the study’s aims. Nonetheless, only one study included in this review reported data on the species, sex, age, weight, body condition, housing size, housing location, type of food, provision of water, and provision of enrichment.

Surprisingly, even within the overall low levels of reporting quality, there were significant differences in reporting quality based on the taxonomic class of the study animals, with studies on mammals reporting several experimental data elements at significantly higher rates than were reported in studies on birds and on reptiles. As an example, compare the differences in the reporting of sex and type of food by class of study species: while 80% of studies on mammals reported the sex of at least one study animal, only 43% of studies on birds and 43% of studies on reptiles reported the sex of at least one study animal. Similarly, while 58% of studies on mammals reported the type of food provided, only 29% of studies on birds and 29% of studies on reptiles reported such data. While the differences in the reporting of sex could be based on the greater challenge of sexing birds and reptiles than sexing mammals, there is no reason the reporting of animal husbandry practices such as the type of food provided should be influenced by whether an animal was a bird, a mammal, or a reptile.

### 4.3. Animal welfare

Even though we specifically searched for studies that included animal welfare type terminology in their titles, abstracts, or keywords, we found limited evidence in the scientific literature on wildlife rescue supporting any focus on animal welfare. While studies that explicitly included the word ‘welfare’ in the main body of the study had higher reporting levels across multiple elements than studies that did not include the word ‘welfare’, even these reporting levels were low. Concerningly, since 2011, the reporting levels across every single experimental animal and housing and husbandry element except housing location have decreased.

### 4.4. Conservation implications

Although our review was primarily focused on welfare, the results of our study also have significant implications for wildlife conservation. Many wildlife enter the rescue system due to human-related causes (e.g. vehicular collision) (Adhikari et al. 2022) and, of the minority that survive to release, post-release outcomes are often poor (Cope et al. 2022). Improving the practice of wildlife rescue could generate population-level conservation benefits, including for threatened species such as the Little Brown Bat (*Myotis lucifugus)* (Paterson et al. 2021). However, key to improving the practice is researchers utilising rigorous methodological designs (Cope et al. 2022; Lane et al. 2024) and reporting the types of data shown to impact outcomes (Cope et al. 2022), the absence of which is detailed in this review.

### 4.5. Geographic and taxonomic biases

Although wildlife rescue is a global practice, the scientific literature we reviewed skewed heavily towards the USA and Australia, with more study sites located in those two countries than all other countries combined. While this skewing could be a result of our English-language criterion, there are currently over 50 countries and territories where English is an official language (CIA n.d.). Nonetheless, only seven of the English-official-language countries were represented in our data (Botswana, Cameroon, Canada, India, Ireland, Malawi, and South Africa). Thus, we considered how searching for animal welfare terminology may have impacted the results. Animal welfare science is a relatively new field and we may have inadvertently excluded studies from countries where the usage of animal welfare type terminology is not yet common (Freire and Nicol 2019).

Regarding taxonomic biases, there was expansive breadth but limited depth in the species coverage. Although 184 unique species were included in studies, no species was included in more than eight studies and 108 species were only included in one study. While there were similar numbers of studies of birds and mammals, only seven studies focused on reptiles. This bias was unsurprising though given that previous studies have highlighted taxonomic biases in wildlife rescue (Shine and Koenig 2001; Wolfe et al. 2019).

### 4.6. Barriers to improvement

Improving the practice of wildlife rescue requires access to findings from high-quality research; however, our study revealed three major barriers to this. Firstly, the reviewed studies were published across 66 journals, with 40 journals containing only one study each. This creates a barrier to access as most veterinarians and wildlife rescuers would not have access to even a portion of these journals. Indeed, even we, with the full resources of a university research library, could not readily access all of the published studies. Secondly, as noted earlier, there are limited data available at the species level or, in the case of reptiles, at even the order level. This means that animals are being treated based on methods developed for entirely different species, with unknown, potentially harmful, consequences (Girolamo et al. 2022; Romero et al. 2024). Lastly, and most critically, even if a veterinary professional or wildlife rescuer were to overcome the first two barriers, due to the general lack of methodological rigour (i.e. controls, randomisation, and blinding) and low reporting quality of experimental data, the results in the current literature base need to be viewed with some caution. Given that our methods were highly permissive, in that we counted as ‘reported’ any mention of a reporting element anywhere in a study for even one study animal, we know that even the low reporting levels reported here represent an overly optimistic view of the literature. We were particularly surprised to discover that journals focused (at least by their titles) on veterinary medicine were associated with lower methodological rigour and reporting quality across multiple elements. Perhaps these results reflect the time-burdens placed on veterinarians (Belshaw et al. 2018) or perhaps they reflect a gap in veterinary training on designing and reporting robust interventional studies (Korkeala and Lindström 2009).

What is clear is that much of the research currently presented in veterinary-focused journals lacks the basic data needed for replicability and applicability. While methodologically weak and poorly reported science does not necessarily equate to poor treatment, without methodologically rigorous designs and high-quality reporting, it is not possible to elucidate the characteristics of high-quality treatment.

### 4.7. Limitations

Although we sought to limit reviewer bias and error through the use of double-blinding, there is the possibility that relevant studies might have been excluded and extraction data were missed. However, given our sample size, we do not believe that such errors, if they occurred, would have unduly altered the results. Similarly, while we only analysed studies published in English, we would not expect studies published in other languages to have had such fundamentally different approaches to the publication of their scientific data that it would have significantly impacted our results (Moher et al. 2000). Nonetheless, future research should strive to analyse the scientific literature on wildlife rescue, regardless of the study’s language.

We recognise that some types of study designs (e.g. case studies) do not necessarily contain controls and that the inclusion of such studies might have impacted the calculations of methodological rigour. However, we elected to include all types of studies that involved an intervention, regardless of the study design, in order to accurately assess the depth and breadth of the scientific evidence available.

Our final literature search date was in April 2021 and due to the significant time requirements of double blinded screening and double blinded data extraction, this study only analysed the scientific literature indexed up to that date. While some literature published subsequently may have improved in rigour and quality, our readings outside of those analysed in this review have not led us to believe later literature would have altered our results.

### 4.8. Recommendations

To improve the methodological rigour and the reporting quality of the scientific research on wildlife rescue, we recommend that: (a) training programs for people involved in trialling rescue, rehabilitation, and release interventions incorporate training on methodological rigour and reporting quality alongside training of hands-on skills; (b) animal ethics boards require reporting protocols such as the Guidelines be followed as part of the ethics approval process; (c) people involved in trialling rescue, rehabilitation and release interventions rigorously apply the Guidelines (or similar) to the design, implementation, and publication of their research; and (d) journals publishing interventional animal studies adopt and enforce mandatory reporting protocols such as the Guidelines.

### 4.9. Future research directions

Both the literature on wildlife rescue and the Guidelines present a broad scope of opportunities for future research. For example, building on our focus on methodological rigour and reporting of universal animal welfare data, researchers could analyse the extent to which organisations are reporting use of animal ethics processes (item 14) and adverse events (item 16b) to explore if there are any variations in outcomes when such information is reported (item 10).

Expanding from a focus on animal welfare to a one welfare framework, the literature on wildlife rescue provides excellent opportunities to explore the wildlife–human health interactions. For example, a systematic review of the zoonotic disease potential of wildlife rescue could assist with identifying zoonotic crossover events (Mathews et al. 2023; Tan et al. 2024). Similarly, a scoping review and evidence map of housing, husbandry, and quarantine practices could be used to identify the procedures being utilised to protect both rescued wildlife and human workers (Bjork et al. 2024).

## 5. Conclusions

1. The existing literature in the field of wildlife rescue, rehabilitation, and release is primarily descriptive, with limited methodological rigour and limited reporting of basic experimental data.
2. There is breadth but limited depth in the species coverage of the literature, with far greater coverage of birds and mammals than of reptiles.
3. There are marked differences in methodological rigour and reporting quality based on whether a journal is focused on veterinary medicine, the class of the study species, and the simple inclusion of the word ‘welfare’.
4. The rates of studies reporting basic data on experimental animals and housing and husbandry declined across nine out of ten elements between 1981–2021. Most notably, since 2010, barely 20% of studies reported any form of enrichment and fewer than 30% of studies reported body condition, housing size, or the provision of water.
5. The risk of the extant scientific literature on wildlife rescue is that veterinarians and wildlife rescuers, faced with limited access to species-specific, robust, high-quality research, may have to use evidentiarily weak, potentially harmful treatments on rescued wildlife.
6. To improve the methodological rigour and the reporting quality of the scientific research on wildlife rescue, we recommend that researchers rigorously apply the ARRIVE guidelines 2.0 (or similar) to the design, implementation, and publication of their research.

## Supplementary Material

Supplemental Material

## Data Availability

All data used for analyses are available at 10.6084/m9.figshare.26863558.
