# Supplementary material for: Methodological rigour and reporting quality of the literature on wildlife rescue, rehabilitation, and release: a global systematic review
Source: Vet Q. 2025 Apr 7;45(1):1–12. doi: 10.1080/01652176.2025.2478138 (PMC11980188; doi:10.1080/01652176.2025.2478138)
Supplement: Supplemental Material [file TVEQ_A_2478138_SM5548.zip › Suppl_Mat/241116_Appendix_A_PRISMA_2020_checklist.docx]

| **Section and Topic** | **Item #** | **Checklist item** | **Location where item is reported** |
| --- | --- | --- | --- |
| **TITLE** | | |  |
| Title | 1 | Identify the report as a systematic review. | Title |
| **ABSTRACT** | | |  |
| Abstract | 2 | See the PRISMA 2020 for Abstracts checklist. | Veterinary Quarterly’s author guidelines are for an unstructured, 200-word maximum abstract. For that reason, the abstract has been limited to background, objectives, limited inclusion criteria, and results. |
| **INTRODUCTION** | | |  |
| Rationale | 3 | Describe the rationale for the review in the context of existing knowledge. | 1.Introduction |
| Objectives | 4 | Provide an explicit statement of the objective(s) or question(s) the review addresses. | 1.Introduction |
| **METHODS** | | |  |
| Eligibility criteria | 5 | Specify the inclusion and exclusion criteria for the review and how studies were grouped for the syntheses. | 2.2.1. Exclusion criteria, 2.2.2. Inclusion criteria,  2.4. Statistical analyses,  Table 2 |
| Information sources | 6 | Specify all databases, registers, websites, organisations, reference lists and other sources searched or consulted to identify studies. Specify the date when each source was last searched or consulted. | 2.1.1. Preliminary searches,  2.1.2. Final search |
| Search strategy | 7 | Present the full search strategies for all databases, registers and websites, including any filters and limits used. | Appendix B |
| Selection process | 8 | Specify the methods used to decide whether a study met the inclusion criteria of the review, including how many reviewers screened each record and each report retrieved, whether they worked independently, and if applicable, details of automation tools used in the process. | 2.2. Screening,  2.2.1. Exclusion criteria, 2.2.2. Inclusion criteria |
| Data collection process | 9 | Specify the methods used to collect data from reports, including how many reviewers collected data from each report, whether they worked independently, any processes for obtaining or confirming data from study investigators, and if applicable, details of automation tools used in the process. | 2.2. Screening  2.3. Data extraction |
| Data items | 10a | List and define all outcomes for which data were sought. Specify whether all results that were compatible with each outcome domain in each study were sought (e.g. for all measures, time points, analyses), and if not, the methods used to decide which results to collect. | As our study focused on methodological rigor and reporting quality and not specific interventions and outcomes, we did extract outcome data from the individual studies.  The types of data we extracted for our analyses of methodological rigor and reporting quality are described in:  2.3. Data extraction,  Table 1,  Table 2,  2.4. Statistical analyses. |
|  | 10b | List and define all other variables for which data were sought (e.g. participant and intervention characteristics, funding sources). Describe any assumptions made about any missing or unclear information. | 2.3. Data extraction, Table 1,  Table 2,  2.4. Statistical analyses. |
| Study risk of bias assessment | 11 | Specify the methods used to assess risk of bias in the included studies, including details of the tool(s) used, how many reviewers assessed each study and whether they worked independently, and if applicable, details of automation tools used in the process. | As we included any primary study of any design that involved an intervention from any time across any species and we did not compare any specific methods or outcomes, we did not conduct risk of bias assessment for the included studies. However, as described in 2.3. Data extraction, 2-3 blinded extractors did analyse three key components of risk of bias (blinding, randomisation, and use of a control) for every reviewed study. |
| Effect measures | 12 | Specify for each outcome the effect measure(s) (e.g. risk ratio, mean difference) used in the synthesis or presentation of results. | As our study focused on methodological rigor and reporting quality and not specific interventions and outcomes, we did not use any effect measure(s) to synthesise or report the results of the individual studies.  The methods we used to tabulate, calculate, and visualise our analyses of methodological rigor and reporting quality are described in:  2.3. Data extraction, and  2.4. Statistical analyses. |
| Synthesis methods | 13a | Describe the processes used to decide which studies were eligible for each synthesis (e.g. tabulating the study intervention characteristics and comparing against the planned groups for each synthesis (item #5)). | As our study focused on methodological rigor and reporting quality and we did not analyse specific outcomes, we neither included synthesis of individual study results nor excluded any eligible studies from analyses based on their results.  The methods we used to screen and group our analyses of methodological rigor and reporting quality are described in:  2.2. Screening,  2.2.1. Exclusion criteria, 2.2.2. Inclusion criteria,  2.3. Data extraction, and  2.4. Statistical analyses. |
|  | 13b | Describe any methods required to prepare the data for presentation or synthesis, such as handling of missing summary statistics, or data conversions. | As our study focused on methodological rigor and reporting quality and not specific interventions and outcomes, we did not prepare the results of the individual studies for presentation or synthesis.  The methods we used to prepare the results of our analyses of methodological rigor and reporting quality are described in:  2.3. Data extraction, and  2.4. Statistical analyses. |
|  | 13c | Describe any methods used to tabulate or visually display results of individual studies and syntheses. | As our study focused on methodological rigor and reporting quality and not specific interventions and outcomes, we did not tabulate or visualise the results of the individual studies.  The methods we used to tabulate and visualise our analyses of methodological rigor and reporting quality are described in:  2.4. Statistical analyses. |
|  | 13d | Describe any methods used to synthesize results and provide a rationale for the choice(s). If meta-analysis was performed, describe the model(s), method(s) to identify the presence and extent of statistical heterogeneity, and software package(s) used. | As our study focused on methodological rigor and reporting quality and not specific interventions and outcomes, we did not synthesise the results of the individual studies.  The statistical methods we used to conduct our analyses of methodological rigor and reporting quality are described in:  2.4. Statistical analyses, and  Table 2. |
|  | 13e | Describe any methods used to explore possible causes of heterogeneity among study results (e.g. subgroup analysis, meta-regression). | As our study focused on methodological rigor and reporting quality and not specific interventions and outcomes, we did explore any possible causes of heterogeneity in the results of the individual studies.  The statistical methods to compare any heterogeneity of results of our analyses of methodological rigor and reporting quality are described in:  2.4. Statistical analyses, and  Table 2. |
|  | 13f | Describe any sensitivity analyses conducted to assess robustness of the synthesized results. | As our study focused on methodological rigor and reporting quality and not specific interventions and outcomes, we did conduct any sensitivity analyses of the results of the individual studies. |
| Reporting bias assessment | 14 | Describe any methods used to assess risk of bias due to missing results in a synthesis (arising from reporting biases). | As we included any primary study of any design that involved an intervention from any time across any species and we did not compare any specific methods or outcomes, we did not conduct a risk of bias assessment due to missing results. We recognise studies that produced negative results may not published but that such studies could potentially have had different levels of methodological rigor and reporting quality. However, our aim was to analyse the extant literature and the evidence that is available for wildlife rescue practitioners and veterinary professionals.  As described in 2.3. Data extraction, 2-3 blinded extractors did analyse three key components of risk of bias (blinding, randomisation, and use of a control) for every reviewed study. |
| Certainty assessment | 15 | Describe any methods used to assess certainty (or confidence) in the body of evidence for an outcome. | As our study focused on methodological rigor and reporting quality and not specific interventions and outcomes, we did conduct any certainty / confidence analyses of the results of the individual studies.  As described in 2.3. Data extraction, 2-3 blinded extractors did analyse three key components of risk of bias (blinding, randomisation, and use of a control) for every article. |
| **RESULTS** | | |  |
| Study selection | 16a | Describe the results of the search and selection process, from the number of records identified in the search to the number of studies included in the review, ideally using a flow diagram. | 3.1. Search and screening results,  3.1.1. Title and abstract screening,  3.1.2. Full text screening  Figure 1,  Appendix B |
|  | 16b | Cite studies that might appear to meet the inclusion criteria, but which were excluded, and explain why they were excluded. | We did not exclude any studies that met the inclusion criteria.  Inclusion and exclusion data are shown in Figure 1, and all included and excluded studies are provided in Appendix C. |
| Study characteristics | 17 | Cite each included study and present its characteristics. | Appendix C |
| Risk of bias in studies | 18 | Present assessments of risk of bias for each included study. | As we included any primary study of any design that involved an intervention from any time across any species and we did not compare any specific methods or outcomes, we did not report risk of bias assessment for the included studies.  However, as described in 2.3. Data extraction, 2-3 blinded extractors did analyse three key components of risk of bias (blinding, randomisation, and use of a control) for each included study. The results for each individual study are reported in Appendix C. |
| Results of individual studies | 19 | For all outcomes, present, for each study: (a) summary statistics for each group (where appropriate) and (b) an effect estimate and its precision (e.g. confidence/credible interval), ideally using structured tables or plots. | As our study focused on methodological rigor and reporting quality and not specific interventions and outcomes, we did report outcome data from the individual studies.  The data we extracted for our analyses of methodological rigor and reporting quality are shown in:  3.2.1. Aim (i) quantify the robustness of the scientific literature on wildlife rescue,  Figure 2,  3.2.2. Aim (ii) quantify the reporting quality of experimental data,  Figure 3,  and  Appendix C.  The summary and descriptive statistics for subgroupings are shown in:  3.2.3 Aim (iii) tests for associations between aims (i), (ii), and study characteristics,  Table 3, and  Table 4. |
| Results of syntheses | 20a | For each synthesis, briefly summarise the characteristics and risk of bias among contributing studies. | As we included any primary study of any design that involved an intervention from any time across any species and we did not compare any specific methods or outcomes, we did not report any risk of bias assessment for the included studies. However, the reporting of randomisation, blinding, and use of control reported in:  3.2.1. Aim (i) quantify the methodological rigor of the scientific literature on wildlife rescue,  Figure 2, and  Appendix C. |
|  | 20b | Present results of all statistical syntheses conducted. If meta-analysis was done, present for each the summary estimate and its precision (e.g. confidence/credible interval) and measures of statistical heterogeneity. If comparing groups, describe the direction of the effect. | As our study focused on methodological rigor and reporting quality and not specific interventions and outcomes, we did not report any statistical synthesis of the results of the individual studies.  The results of the statistical analyses of methodological rigor and reporting quality are reported in:  3.2.1. Aim (i) quantify the robustness of the scientific literature on wildlife rescue,  Figure 2,  3.2.2. Aim (ii) quantify the reporting quality of experimental data,  Figure 3,  3.2.3 Aim (iii) tests for associations between aims (i), (ii), and study characteristics,  and  Appendix C.  The summary and descriptive statistics for subgroupings are shown in  Table 3, and  Table 4. |
|  | 20c | Present results of all investigations of possible causes of heterogeneity among study results. | As our study focused on methodological rigor and reporting quality and not specific interventions and outcomes, we did report any possible causes of heterogeneity in the results of the individual studies.  The results of the statistical methods we used analyse heterogeneity in methodological rigor and reporting quality are reported in:  3.2.1. Aim (i) quantify the robustness of the scientific literature on wildlife rescue,  Figure 2,  3.2.2. Aim (ii) quantify the reporting quality of experimental data,  Figure 3,  3.2.3 Aim (iii) tests for associations between aims (i), (ii), and study characteristics,  Table 3,  Table 4,  and  Appendix C. |
|  | 20d | Present results of all sensitivity analyses conducted to assess the robustness of the synthesized results. | As our study focused on methodological rigor and reporting quality and not specific interventions and outcomes, we did report any sensitivity analyses of the results of the individual studies. |
| Reporting biases | 21 | Present assessments of risk of bias due to missing results (arising from reporting biases) for each synthesis assessed. | As we included any primary study of any design that involved an intervention from any time across any species and we did not compare any specific methods or outcomes, we did not report a risk of bias assessment due to missing results. We recognise studies that produced negative results may not published but that such studies could potentially have had different levels of methodological rigor and reporting quality. However, our aim was to analyse the extant literature and the evidence that is available for wildlife rescue practitioners and veterinary professionals.  The data we extracted for our analyses of methodological rigor are reported in:  3.2.1. Aim (i) quantify the robustness of the scientific literature on wildlife rescue,  Figure 2,  Table 4, and  Appendix C. |
| Certainty of evidence | 22 | Present assessments of certainty (or confidence) in the body of evidence for each outcome assessed. | As we included any primary study of any design that involved an intervention from any time across any species and we did not compare any specific methods or outcomes, we did not report any certainty or confidence assessments on the individual studies.  The data we extracted for our analyses of methodological rigor are reported in:  3.2.1. Aim (i) quantify the robustness of the scientific literature on wildlife rescue,  Figure 2,  Table 4, and  Appendix C. |
| **DISCUSSION** | | |  |
| Discussion | 23a | Provide a general interpretation of the results in the context of other evidence. | 4. Discussion,  4.1. Methodological rigor,  4.2. Reporting quality,  4.3. Animal welfare, |
|  | 23b | Discuss any limitations of the evidence included in the review. | 4.4 Geographic and taxonomic biases,  4.5 Barriers to improvement, and  4.6 Limitations |
|  | 23c | Discuss any limitations of the review processes used. | 4.6 Limitations |
|  | 23d | Discuss implications of the results for practice, policy, and future research. | 4.1 Methodological rigor,  4.2 Reporting Quality,  4.3 Animal welfare,  4.4 Geographic and taxonomic biases,  4.5 Barrier to improvement,  4.6 Limitations,  4.7 Recommendations,  4.8 Future research directions, and  5. Conclusions, specifically 5.5 and 5.6 |
| **OTHER INFORMATION** | | |  |
| Registration and protocol | 24a | Provide registration information for the review, including register name and registration number, or state that the review was not registered. | 2. Methods |
|  | 24b | Indicate where the review protocol can be accessed, or state that a protocol was not prepared. | 2. Methods |
|  | 24c | Describe and explain any amendments to information provided at registration or in the protocol. | 2. Methods |
| Support | 25 | Describe sources of financial or non-financial support for the review, and the role of the funders or sponsors in the review. | 6. Acknowledgement |
| Competing interests | 26 | Declare any competing interests of review authors. | 8. Competing Interests |
| Availability of data, code and other materials | 27 | Report which of the following are publicly available and where they can be found: template data collection forms; data extracted from included studies; data used for all analyses; analytic code; any other materials used in the review. | 9. Data availability |

*From:*  Page MJ, McKenzie JE, Bossuyt PM, Boutron I, Hoffmann TC, Mulrow CD, et al. The PRISMA 2020 statement: an updated guideline for reporting systematic reviews. BMJ 2021;372:n71. doi: 10.1136/bmj.n71
