# Supplementary material for: Methodological rigour and reporting quality of the literature on wildlife rescue, rehabilitation, and release: a global systematic review
Source: Vet Q. 2025 Apr 7;45(1):1–12. doi: 10.1080/01652176.2025.2478138 (PMC11980188; doi:10.1080/01652176.2025.2478138)
Supplement: Supplemental Material [file TVEQ_A_2478138_SM5548.zip › Suppl_Mat/241116_Appendix_B_search_terms.docx]

Appendix B: Search terms and results

The following are the search syntaxes used to search SCOPUS and Web of Science on 8 April 2021. The differences in the syntax requirements between SCOPUS and Web of Science necessitated the different search approaches.

**Database: SCOPUS**

| **Advanced search query** | **Number of results returned** |
| --- | --- |
| ( ( TITLE-ABS-KEY ( wildlife OR mammal OR bird OR amphibian OR reptile OR oiled ) W/1 ( centre OR shelter OR clinic OR hospital OR medical OR carer ) ) OR ( TITLE-ABS-KEY ( wildlife OR mammal OR bird OR amphibian OR reptile OR oiled ) W/5 ( rescu* OR rehab* OR post-releas* OR orphan* ) ) ) AND ( TITLE-ABS ( analgesi* OR anesthes* OR euthanas* OR improv* OR handl* OR hous* OR husbandry OR cag* OR non-invasive OR monitor* OR behav* OR "positive reinforcement" OR welfare OR adverse OR pain OR suffer OR stress OR distress OR harm OR mortality OR outcome ) ) AND ( LIMIT-TO ( DOCTYPE , "ar" ) OR LIMIT-TO ( DOCTYPE , "re" ) ) | 798 |

**Database: Web of Science**

| **Search number** | **Advanced search query** | **Indexes selected** | **Number of results returned** |
| --- | --- | --- | --- |
| #1 | (TS=(wildlife NEAR/1 (centre OR shelter OR clinic OR hospital OR medical OR carer) )) AND DOCUMENT TYPES: (Article OR Review) | Indexes=SCI-EXPANDED, SSCI, A&HCI, CPCI-S, CPCI-SSH, BKCI-S, BKCI-SSH, ESCI, CCR-EXPANDED, IC Timespan=All years | 629 |
| #2 | (TS=(mammal NEAR/1 (centre OR shelter OR clinic OR hospital OR medical OR carer) )) AND DOCUMENT TYPES: (Article OR Review) | Indexes=SCI-EXPANDED, SSCI, A&HCI, CPCI-S, CPCI-SSH, BKCI-S, BKCI-SSH, ESCI, CCR-EXPANDED, IC Timespan=All years | 92 |
| #3 | (TS=(bird NEAR/1 (centre OR shelter OR clinic OR hospital OR medical OR carer) )) AND DOCUMENT TYPES: (Article OR Review) | Indexes=SCI-EXPANDED, SSCI, A&HCI, CPCI-S, CPCI-SSH, BKCI-S, BKCI-SSH, ESCI, CCR-EXPANDED, IC Timespan=All years | 134 |
| #4 | (TS=(amphibian NEAR/1 (centre OR shelter OR clinic OR hospital OR medical OR carer) )) AND DOCUMENT TYPES: (Article OR Review) | Indexes=SCI-EXPANDED, SSCI, A&HCI, CPCI-S, CPCI-SSH, BKCI-S, BKCI-SSH, ESCI, CCR-EXPANDED, IC Timespan=All years | 29 |
| #5 | (TS=(reptile NEAR/1 (centre OR shelter OR clinic OR hospital OR medical OR carer) )) AND DOCUMENT TYPES: (Article OR Review) | Indexes=SCI-EXPANDED, SSCI, A&HCI, CPCI-S, CPCI-SSH, BKCI-S, BKCI-SSH, ESCI, CCR-EXPANDED, IC Timespan=All years | 17 |
| #6 | (TS=(oiled NEAR/1 (centre OR shelter OR clinic OR hospital OR medical OR carer) )) AND DOCUMENT TYPES: (Article OR Review) | Indexes=SCI-EXPANDED, SSCI, A&HCI, CPCI-S, CPCI-SSH, BKCI-S, BKCI-SSH, ESCI, CCR-EXPANDED, IC Timespan=All years | 373 |
| #7 | (TS=(wildlife NEAR/5 (rescu* OR rehab* OR post-releas* OR orphan*) )) AND DOCUMENT TYPES: (Article OR Review) | Indexes=SCI-EXPANDED, SSCI, A&HCI, CPCI-S, CPCI-SSH, BKCI-S, BKCI-SSH, ESCI, CCR-EXPANDED, IC Timespan=All years | 531 |
| #8 | (TS=(mammal NEAR/5 (rescu* OR rehab* OR post-releas* OR orphan*) )) AND DOCUMENT TYPES: (Article OR Review) | Indexes=SCI-EXPANDED, SSCI, A&HCI, CPCI-S, CPCI-SSH, BKCI-S, BKCI-SSH, ESCI, CCR-EXPANDED, IC Timespan=All years | 93 |
| #9 | TS=(bird NEAR/5 (rescu* OR rehab* OR post-releas* OR orphan*) )) AND DOCUMENT TYPES: (Article OR Review) | Indexes=SCI-EXPANDED, SSCI, A&HCI, CPCI-S, CPCI-SSH, BKCI-S, BKCI-SSH, ESCI, CCR-EXPANDED, IC Timespan=All years | 260 |
| #10 | TS=(amphibian NEAR/5 (rescu* OR rehab* OR post-releas* OR orphan*) )) AND DOCUMENT TYPES: (Article OR Review) | Indexes=SCI-EXPANDED, SSCI, A&HCI, CPCI-S, CPCI-SSH, BKCI-S, BKCI-SSH, ESCI, CCR-EXPANDED, IC Timespan=All years | 19 |
| #11 | (TS=(reptile NEAR/5 (rescu* OR rehab* OR post-releas* OR orphan*) )) AND DOCUMENT TYPES: (Article OR Review) | Indexes=SCI-EXPANDED, SSCI, A&HCI, CPCI-S, CPCI-SSH, BKCI-S, BKCI-SSH, ESCI, CCR-EXPANDED, IC Timespan=All years | 14 |
| #12 | (TS=(oiled NEAR/5 (rescu* OR rehab* OR post-releas* OR orphan*) )) AND DOCUMENT TYPES: (Article OR Review) | Indexes=SCI-EXPANDED, SSCI, A&HCI, CPCI-S, CPCI-SSH, BKCI-S, BKCI-SSH, ESCI, CCR-EXPANDED, IC Timespan=All years | 269 |
| #13 | #12 OR #11 OR #10 OR #9 OR #8 OR #7 OR #6 OR #5 OR #4 OR #3 OR #2 OR #1 | Indexes=SCI-EXPANDED, SSCI, A&HCI, CPCI-S, CPCI-SSH, BKCI-S, BKCI-SSH, ESCI, CCR-EXPANDED, IC Timespan=All years | 2,059 |
| #14 | (AB=( analgesi* OR anesthes* OR euthanas* OR improv* OR handl* OR hous* OR husbandry OR cag* OR non-invasive OR monitor* OR behav* OR "positive reinforcement" OR welfare OR adverse OR pain OR suffer OR stress OR distress OR harm OR mortality OR outcome) ) AND DOCUMENT TYPES: (Article OR Review) | Indexes=SCI-EXPANDED, SSCI, A&HCI, CPCI-S, CPCI-SSH, BKCI-S, BKCI-SSH, ESCI, CCR-EXPANDED, IC Timespan=All years | 11,197,818 |
| #15 | (TI=(analgesi* OR anesthes* OR euthanas* OR improv* OR handl* OR hous* OR husbandry OR cag* OR non-invasive OR monitor* OR behav* OR "positive reinforcement" OR welfare OR adverse OR pain OR suffer OR stress OR distress OR harm OR mortality OR outcome) ) AND DOCUMENT TYPES: (Article OR Review) | Indexes=SCI-EXPANDED, SSCI, A&HCI, CPCI-S, CPCI-SSH, BKCI-S, BKCI-SSH, ESCI, CCR-EXPANDED, IC Timespan=All years | 2,697,053 |
| #16 | (#15 OR #14) AND DOCUMENT TYPES: (Article OR Review) | Indexes=SCI-EXPANDED, SSCI, A&HCI, CPCI-S, CPCI-SSH, BKCI-S, BKCI-SSH, ESCI, CCR-EXPANDED, IC Timespan=All years | 11,975,401 |
| #17 | (#16 AND #13) AND DOCUMENT TYPES: (Article OR Review) | Indexes=SCI-EXPANDED, SSCI, A&HCI, CPCI-S, CPCI-SSH, BKCI-S, BKCI-SSH, ESCI, CCR-EXPANDED, IC Timespan=All years | 941 |
