# Supplementary material for: Methodological rigour and reporting quality of the literature on wildlife rescue, rehabilitation, and release: a global systematic review
Source: Vet Q. 2025 Apr 7;45(1):1–12. doi: 10.1080/01652176.2025.2478138 (PMC11980188; doi:10.1080/01652176.2025.2478138)
Supplement: Supplemental Material [file TVEQ_A_2478138_SM5548.zip › Suppl_Mat/241116_Appendix_C_link_to_Figshare.docx]

Appendix C: Data

To download the Excel file containing all of the data used in our analyses, please go to:

<https://figshare.com/s/79c87e48245d8eb7a881>.
